# Powder and Nanotubes Titania Modified by Dye Sensitization as Photocatalysts for the Organic Pollutants Elimination

**DOI:** 10.3390/nano9040517

**Published:** 2019-04-02

**Authors:** Julie J. Murcia, Elsa G. Ávila-Martínez, Hugo Rojas, Jairo Cubillos, Svetlana Ivanova, Anna Penkova, Oscar H. Laguna

**Affiliations:** 1 Grupo de Catálisis de la Universidad Pedagógica y Tecnológica de Colombia, Avenida Central del Norte 39-115, Tunja 150003, Colombia; julie.murcia@uptc.edu.co (J.J.M.); elsa.avila@uptc.edu.co (E.G.Á.-M.); hugo.rojas@uptc.edu.co (H.R.); jairo.cubillos@uptc.edu.co (J.C.); 2 Departamento de Química Inorgánica / Universidad de Sevilla and Instituto de Ciencia de Materiales de Sevilla, Centro Mixto Universidad de Sevilla—CSIC, Avenida Américo Vespucio, 49, 41092 Seville, Spain; svetlana@icmse.csic.es (S.I.); anna@icmse.csic.es (A.P.)

**Keywords:** TiO_2_ powders, TiO_2_ nanotubes, dye-sensitized TiO_2_, photocatalysis

## Abstract

In this study, titanium dioxide powder obtained by the sol-gel method and TiO_2_ nanotubes, were prepared. In order to increase the TiO_2_ photoactivity, the powders and nanotubes obtained were modified by dye sensitization treatment during the oxide synthesis. The sensitizers applied were Quinizarin (Q) and Zinc protoporphyrin (P). The materials synthesized were extensively characterized and it was found that the dye sensitization treatment leads to modify the optical and surface properties of Titania. It was also found that the effectiveness of the dye-sensitized catalysts in the phenol and methyl orange (MO) photodegradation strongly depends on the dye sensitizer employed. Thus, the highest degradation rate for MO was obtained over the conventional Q-TiO_2_ photocatalyst. In the case of the nanotubes series, the most effective photocatalyst in the MO degradation was based on TiO_2_-nanotubes sensitized with the dye protoporfirin (ZnP). Selected catalysts were also tested in the phenol and MO photodegradation under visible light and it was observed that these samples are also active under this radiation.

## 1. Introduction

As a result of the industrial and human activities, large volumes of wastewater containing different pollutants are poured every year, significantly affecting the environmental stability. Therefore, for the treatment of urban and industrial wastewater, different alternatives have been employed. Despite this, in most of cases, pollutants are recalcitrant, non-biodegradable and often the formation of more toxic products during the treatment is noticeable [1,2]. Phenolic compounds and dyestuffs are common organic pollutants in wastewater, which have proven to be hard to remove. With this outlook, currently, it is necessary to search for more suitable and effective methods for environmental pollutants removal.

TiO_2_ photocatalysis has been successfully applied as an eco-friendly and efficient alternative in the treatment of wastewater sources and polluted atmospheres [3,4,5,6,7,8,9,10,11,12]. TiO_2_ has been considered as the best photocatalyst, the photoactivity of this oxide depends not only on its physicochemical properties but also on its structure.

TiO_2_-based nanotubes production has been extensively studied by Kasuga et al. [13,14]. Since 1998, these authors have demonstrated that these nanotubes have great potential for use in the preparation of catalysts, adsorbents, and deodorants with high activities because their specific surface area is greatly increased. TiO_2_-based nanotubes have attracted great interest to be used as photocatalysts in environmental applications [15,16,17,18,19,20]. Currently, the advances in the study of nanotubes have been focused in self-organized anodic TiO_2_ nanotube layers and the use of atomic layer deposition for the functionalization of these layers. By using this technique, important advances in the improvement of physicochemical, photoelectrochemical and photocatalytic properties of Titania have been achieved [21]. Additionally, other photocatalysts based on nanowire arrays like structures or heterostructures have also been studied [22,23,24].

In order to modify TiO_2_ structure, the preparation of layered titanates have also received great attention, mainly due to the high ability to ion exchange/intercalation reactions and potential applications in the synthesis of new nanomaterials. By using bulk organic molecules in intercalation processes, it is possible to produce TiO_2_ single sheets with 2D morphology [20].

TiO_2_ presents some disadvantages, such as higher recombination rate of the photogenerated charges and the largest band gap value. Consequently, in order to solve these problems and for increasing the TiO_2_ photoactivity, many strategies have been employed, where usually noble metal addition is a good alternative to reduce the recombination [25,26]. Currently, the dye sensitization also represents a good way to increase the TiO_2_ photo efficiency in different chemical reactions [27]. The sensitization leads to the increase of visible light adsorption as usually, the sensitizer applied in photocatalytic processes is a chromophore compound, which is anchored to the semiconductor surface. Such dye molecules absorb visible light and excites electrons, and these electrons get transferred to the conduction band in the semiconductor, leading to decrease of its band gap value [28].

According to the scenario presented above, the main objective of this research was to study the effectiveness of photocatalysts based on sol-gel synthesized TiO_2_ powders and TiO_2_ nanotubes, in the phenol and methyl orange photodegradation in the liquid phase. As a strategy to increase the activity of the photocatalysts, these materials were modified by sensitization with different dyes. The dyes selected was also employed as bulky molecules to induce the obtention of layered titanates.

## 2. Materials and Methods

### 2.1. Photocatalysts Preparation

**Figure 1:**
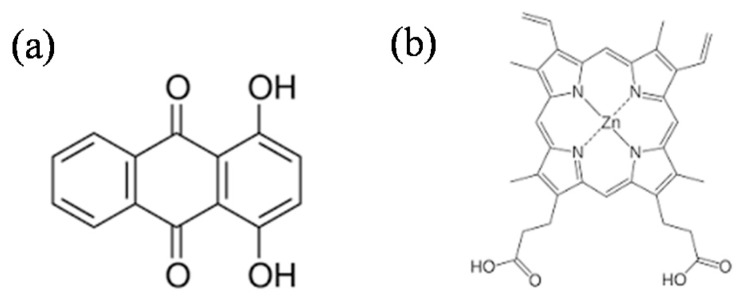
The molecular structure of organic dyes used in the present work as photocatalysts sensitizers. (**a**) Quinizarin and (**b**) Zinc protoporphyrin.

The sol-gel TiO_2_ powder was obtained by controlled hydrolysis of 1 mol of titanium butoxide (IV). As hydrolysis rate controllers, acetic acid (4 mol) and ethanol (5 mol) were added. After homogenization, 8 mol of distilled water was added drop by drop and the hydrolysis maintained under continuous stirring for 3 h. The powders thus obtained were recovered by filtration and dried at 80 °C. Moreover, in order to obtain the sensitized TiO_2_, 5 mmol of quinizarin (Q) or Zinc protoporphyrin (ZnP) were incorporated in the suspension before the hydrolysis step. The samples were labeled as Q and ZnP and Figure 1 shows the structure of the sensitizers molecules. It was previously indicated in the introduction section that in the present work, we attempted to prepare layered titanates by using bulk chemicals for the separation of these layers. In order to achieve this objective, we select two different organic molecules such as Quinizarin and Zinc protoporphyrin. These molecules presented marked differences in size and chemical composition, so it was interesting to study the effect of these molecules in addition to the structure of Titania obtained and also to the photoactivity of this oxide.

In order to convert the as prepared sol-gel TiO_2_ powders into titania nanotubes, the sol-gel product was dispersed in 7 M of NaOH aqueous solution and maintained under stirring for 24 h. After this, a washing with distilled water was carried out and the material thus obtained was dried at 80º C. The obtained samples in the form of nanotubes were labeled as Qa and ZnPa.

The control samples produced without colorants, such as powders TiO_2_ and TiO_2_ nanotubes for the sol-gel received materials and its treatment by NaOH were labeled as LT and TNT, respectively.

### 2.2. Photocatalysts Characterization

The materials synthesized were characterized by means of the different techniques described below.

X-ray diffraction (XRD) analysis was performed on an X’PertPro PANalytical instrument (Malvern, UK). Diffraction patterns were recorded with the Cu Kα radiation (40 mA, 45 kV) over a 2θ-range of 3–60°, and a position-sensitive detector using a step size of 0.05° and a step time of 240 s.

All materials were analyzed by UV-Vis diffuse reflection spectroscopy by using a Varian spectrometer model Cary 100 (Palo Alto, CA, USA) and a BaSO_4_ sphere as the reference. All the spectra were collected in diffuse reflectance mode and transformed to a magnitude proportional to the extinction coefficient through the Kubelka–Munk function.

The textural properties were studied by the means of N_2_ adsorption–desorption measurements at liquid nitrogen temperature. The experiments were carried out on a Micromeritics ASAP 2010 instrument (Norcross, GA, USA). Before analysis, the samples were degassed for 2 h at 150 °C in the vacuum.

All photocatalysts were also evaluated by Transmission Electron Microscopy (TEM) in a Philips CM200 instrument (Amsterdam, The Netherlands). For this analysis, the samples were dispersed in ethanol using the ultrasound and dropped on a carbon grid.

### 2.3. Photocatalytic Tests

The photocatalytic activity of the synthesized catalysts was measured in the phenol and methyl orange (MO) photodegradation reactions. Both processes were carried by using a discontinuous batch system, including a 400 mL pyrex reactor enveloped by an aluminum foil, filled with an aqueous suspension (250 mL) containing 25 ppm of phenol or MO and the photocatalyst (1 g/L). This system was illuminated through a UV-transparent Plexiglas® top window (threshold absorption at 250 nm) by a 300 W Osram Ultra-Vitalux lamp (Munich, Germany) with sun-like radiation spectrum and a main line in the UVA range at 365 nm. The intensity of the incident UV-Visible light on the solution was measured with a Delta OHM photoradiometer HD2102.1 (Caselle di Selvazzano, Padova, Italy), being ca. 120 W/m^2^. The visible photocatalytic experiments were performed by using a polyester UV filter sheet (Edmund Optics, Barrington, NJ, USA) showing 99.9% of absorbance below 400 nm (0.15 W/m^2^ for λ < 400 nm and 150 W/m^2^ for λ > 400 nm). In order to favor the adsorption–desorption equilibrium, prior to the irradiation, the suspension was magnetically stirred for 10 min in the dark. Furthermore, a constant oxygen flow of 25 L/h used as the oxidant was passed through the suspension for improving the homogeneous dispersion of the photocatalyst in the solution. For this purpose, a bubbler tank was used as a source of natural oxygen. All photocatalytic tests started at the natural pH of pollutants solutions which was ca. 6, and the total reaction time was 120 min.

During the phenol and MO photoreactions, samples were collected at different times and analyzed by UV-Visible spectrophotometry, considering the main absorption band observed for these compounds and located at 270 and 465 nm, for phenol and MO, respectively. For these analyses, a Genesys 10UV Thermo Electron instrument (Waltham, MA, USA) was used. Taking into account the Lambert–Beer law which stated that the absorbance is proportional to the concentrations, the evolution of these pollutants concentration as a function of the reaction time was calculated from the calibration curve obtained from the UV-Vis analyzes. The pollutants photodegradation rate was also determined by using Equation (1):

(1) $$v=k\times C_{0}\times V$$

where, *v* = photodegradation rate, *k* = Initial reaction constant, taken from the slope of the graph representing concentration vs. reaction time (s^−1^), *C*_0_ = initial concentration of the substrates (Phenol or MO) (mol/L), *V* = volume of Phenol or MO (L).

Photolysis tests of phenol and MO under UV-Visible light and in the absence of catalyst were carried out. Reproducibility of the measurements was ensured by double testing of selected samples.

## 3. Results and Discussion

### 3.1. Photocatalysts Characterization

**Figure 2:**
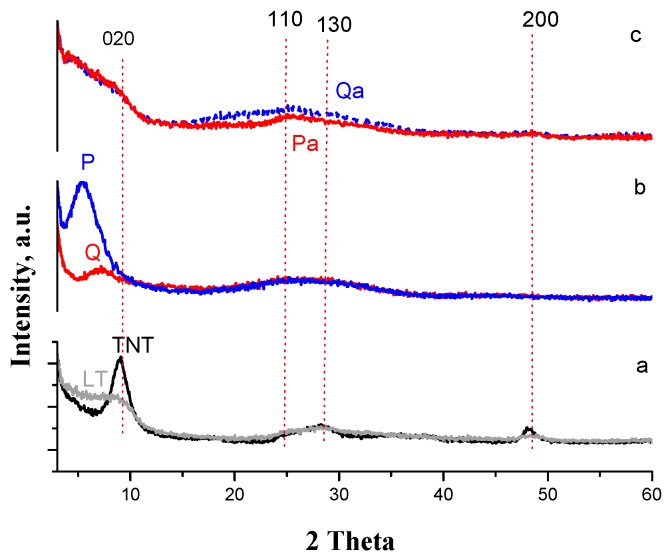
X-ray diffraction patterns of the photocatalysts analyzed. (**a**) TiO_2_ powders and nanotubes without sensitizers; (**b**) TiO_2_ powders sensitized and (**c**) TiO_2_ nanotubes sensitized.

Figure 2 shows the X-ray diffraction patterns of the analyzed photocatalysts. The bare titania materials (Figure 2a) exhibit the presence of low crystalline sol-gel synthesized material. However, the production of lamellar structure can be noticed by the diffraction observed at low 2 theta angles. This diffraction can be ascribed to the presence of (0k0) body-centered orthorhombic titanate lepidocrocite like structure, commonly known as lamellar titanates. This structure was confirmed also by the asymmetric line shape of the reflection tailing toward higher angle diffractions and typical two-dimensional lattice of the layered titanates. Its treatment with NaOH (TNT sample) increases the intensity of the reflection and generates the appearance of diffraction lines associated with the (110), (130) and (200) crystallographic planes, being the last one unequivocal sign of titania nanotubes production [20].

When the colorants are used (Q and ZnP samples, Figure 2b) the same diffractions as for the LT sample can be noticed, although a shift to lower 2 theta angles is observed, suggesting interplanar distance increasing due to the colorants molecule hosting. The interplanar lamellar distance of titania increases with the kinetic diameter of the molecule, following the order of bare titania (LT sample) < Quinizarin (Q) < protoporphyrin (ZnP) sensitized titania materials.

Moreover, after NaOH treatment (Figure 2c), the ZnPa and Qa samples do not present the signal at 2 theta 49 (fingerprint of titania nanotubes), indicating a low degree of titania nanotubes production or the production of nanotubes with very low crystallinity [20]. The latter could be due to an important interaction of the colorants with the titania layer and difficulty to enroll the nanotubes in the presence of bulky organic molecules. Additionally, the NaOH treatment also produces a slight color change in the material due to either the loss of colorant or to the interaction between the Na^+^ with the chromophore molecules.

The Brunauer–Emmett–Teller (BET) specific surface areas of the samples are presented in Table 1. All starting materials (LT, ZnP, and Q) show specific surface areas ranging between 170–200 m^2^/g, which indicates the production of mesoporous materials with average pore size around 5 nm. The NaOH treatment produces important loss of specific area due to the pore shrinking to around 2 nm for all samples (TNT, Qa, ZnPa).

**Figure 3:**
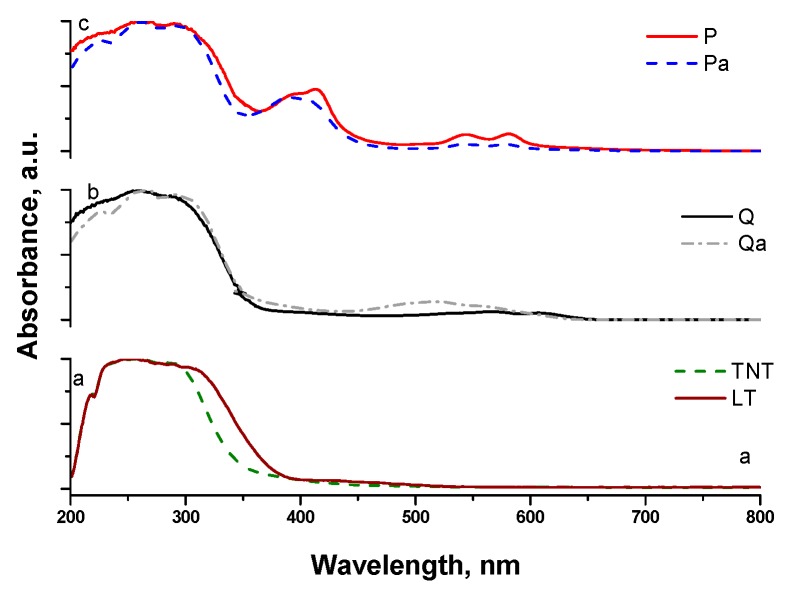
UV-Vis diffuse reflection spectra of the analyzed photocatalysts. (**a**) TiO_2_ powders and nanotubes without sensitizers; (**b**) TiO_2_ sensitized with Quinizarin and (**c**) TiO_2_ sensitized with Zinc protoporphyrin.

The optical properties of the samples were analyzed by means of UV-Vis diffuse reflection spectroscopy and the obtained spectra for all photocatalysts are shown in Figure 3.

The typical band edge of the TiO_2_ semiconductor was observed for all samples near 350 nm. The sensitized materials present also absorption in the visible range (400–700 nm) compared to bare titania materials (Figure 3a). The quinizarin sensitized titania shows maximal absorbance in the 450–570 nm range, which could be shifted as a function of the structuring charge [29,30]. Thus, the neural quinizarin absorbs in 455–496 nm range, monoanionic at 554–569 nm and dianionic at 550 nm. The optical spectra of the Q sample indicate the presence of mono and dianionic quinizarin species interacting with the Ti–O skeleton, whereas the Qa bands shift to the lower wavelength indicating the loss of charge of the quinizarin molecules. The latter could be explained by the presence of Na^+^, which could compensate the charge of the colorant, thus, producing the observed blue shift. However, bands centered at 510 nm indicate either a partial quinizarin charge compensation or the blue shift could be produced by the confinement effect by the nanotube formation.

On the other hand, the metal porphyrins present two types of visible adsorption, one band centered at around 410 nm called Soret band and a group of three to four bands in the 500–650 nm range called Q bands. Two types of bands are observed for our samples (ZnP, ZnPa) regardless of the treatment, indicating the presence of Zn protoporphyrin before and after the NaOH treatment without any significant change in its structure. However, the intensity of the Q bands decreases after NaOH treatment indicating either a lower quantity of colorant or closer colorant to metal energy levels and better orbital mixing due to its confinement in the resulting nanotubes.

**Figure 4:**
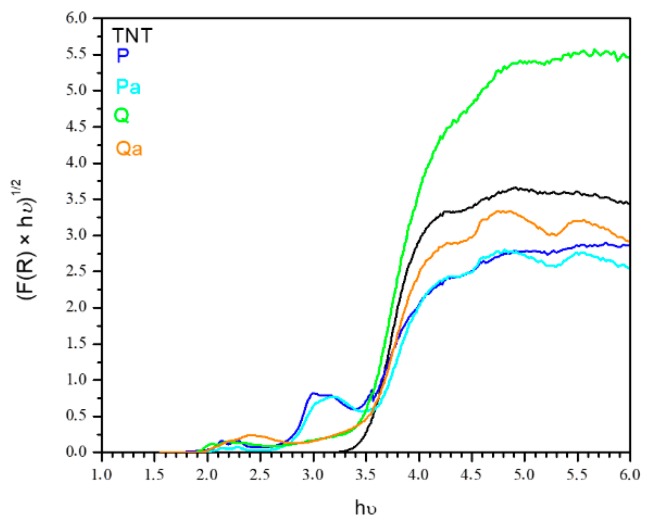
Absorption spectra for the evaluation of band gap energy for photocatalysts analyzed.

The bandgaps energies were also calculated and the results are listed in Table 1. The LT and TNT samples show higher band gap energies 3.4–3.5 eV reduced to 3.2 and 3.3 for the sensitized samples due to the addition of colorant levels in the band gap of the pure titania structure. Figure 4 shows the absorption spectra obtained for the evaluation of band gap energy.

**Figure 5:**
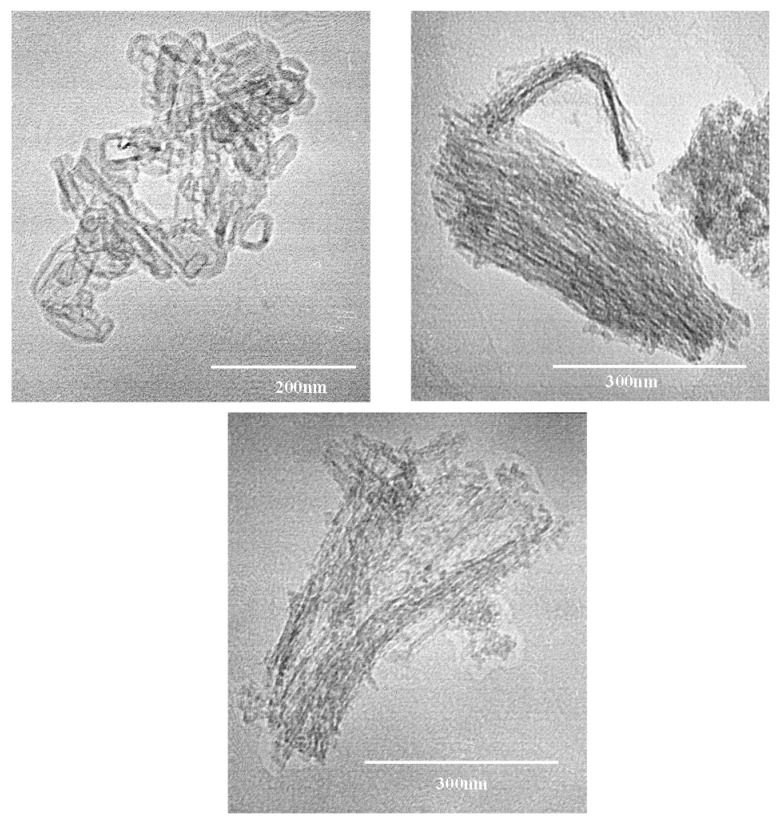
Selected transmission electron microscopy (TEM) micrographs of the TNT sample.

Figure 5 shows selected TEM images for the TNT samples, as it can be seen in these images, there are microchannels in the samples, so, we can observe that the preparation method led us to obtain nanotubes like structures in the materials analyzed.

TiO_2_ as powders and nanotubes were extensively characterized by using the additional instrumental techniques, these results have been previously reported by Ivanova et al. [20].

### 3.2. Photocatalytic Activity

**Figure 6:**
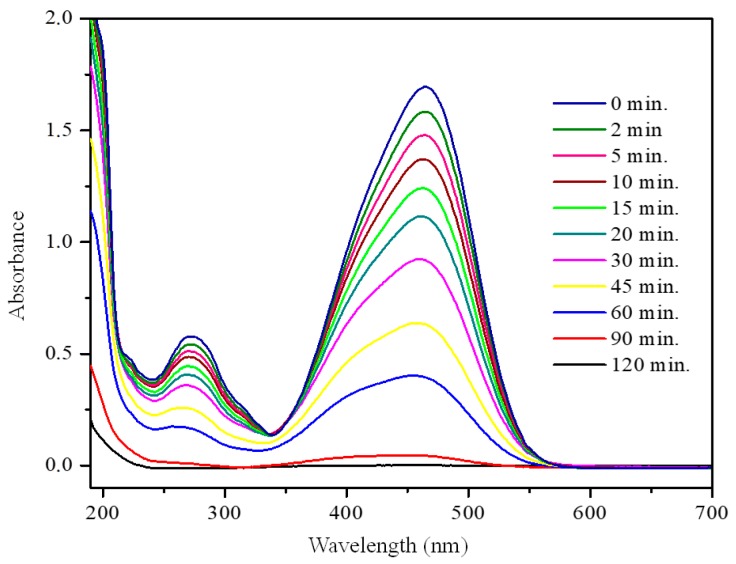
Photocatalytic degradation of methyl orange over commercial TiO_2_ (Aeroxide^®^ TiO_2_ P25 Evonik) as a function of the reaction time.

Figure 6 represents the evolution of methyl orange absorbance during 120 min of photoreaction time by using commercial TiO_2_ P25. As it was previously indicated in Section 2.3, the main absorption band located at 465 nm was employed to estimate the MO concentration. The intensity of this band decreases as the reaction time increases, which indicates the breaking of the chromophore group corresponding to the azo group (N=N) present in the molecule of MO. It is important to note that the absence of new signals in the spectra confirms the degradation of the dye being treated; any reaction intermediate product was detected even after 120 min of reaction time. The UV-Vis spectra obtained with other photocatalysts are not included for the sake of brevity, but similar behavior as described here was observed for P25.

**Figure 7:**
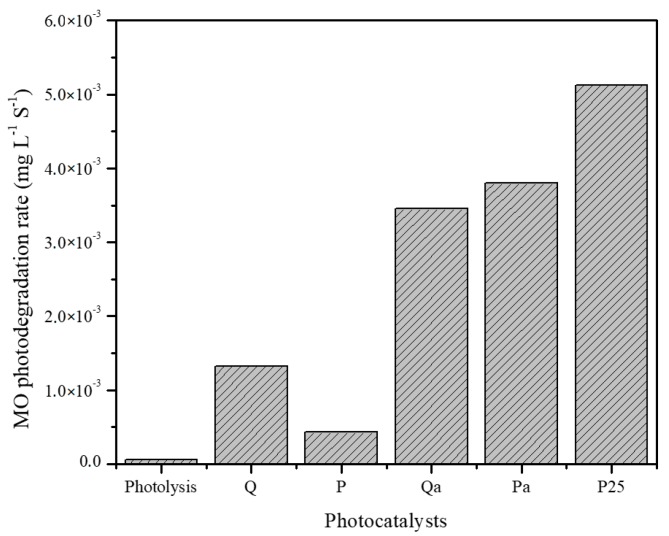
Methyl orange degradation rate obtained by photolysis and photocatalytic treatment by using all the photocatalysts analyzed.

The photocatalytic degradation rate of methyl orange over the analyzed catalysts is represented in Figure 7 and it is observed that the substrate photolysis is negligible, thus, indicating that the presence of a photocatalyst is necessary to induce the highest degradation rate of MO.

In general, in photocatalyst series prepared with conventional sensitized TiO_2_, the highest photodegradation rate for MO was obtained by using quinizarin as sensitizer agent (i.e Q catalyst). This is the result of the highest specific surface area of this material (Table 1), since the substrate-catalyst surface interaction is an important factor influencing the degradation rate. This is because the adsorption of the dye can be improved in the surface of materials with the highest specific surface area, leading to increase in the photodegradation rate. 

Moreover, the catalysts based on TiO_2_-nanotubes sensitized were also evaluated in the MO photodegradation and the results are also included in Figure 7. As it can be observed, the MO degradation rate over the photocatalysts based on TiO_2_-nanotubes sensitized with quinizarin (Qa) and protoporfirin (ZnPa) is significantly higher than the observed over the conventional catalysts. Within the series, the highest dye degradation was achieved by using the ZnPa catalyst, which can be due to the higher *S*_BET_ of this material compared to Qa (Table 1). As the lower specific surface area was measured for the TiO_2_-nanotubes sensitized samples in comparison to the conventional TiO_2_ samples, one can consider its photocatalytic behavior as illogically active. However, the improvement of their activity could be assigned to the presence of sensitizing molecules within the nanotubes, whose electronic properties become promoted by the electron confinement effect in semiconductors [31]. It is also worthy to consider the lower electron-holes recombination rate for this material in comparison to the higher surface materials, since the electrons can initially reach the dye before the conduction band. It is also possible that the sensitized photocatalytic materials simultaneously decompose both methyl orange and the dyes used for the sensitization. In fact, it was observed that after photocatalytic tests, the materials recovered presented a lighter color than they had initially, thus, showing that the sensitizing dye can be modified during photocatalytic reactions.

Commercial TiO_2_ P25 Evonik was used as a reference, and it was observed that in any of the photocatalytic tests, a degradation rate higher than the obtained with this commercial sample (P25) was achieved.

**Figure 8:**
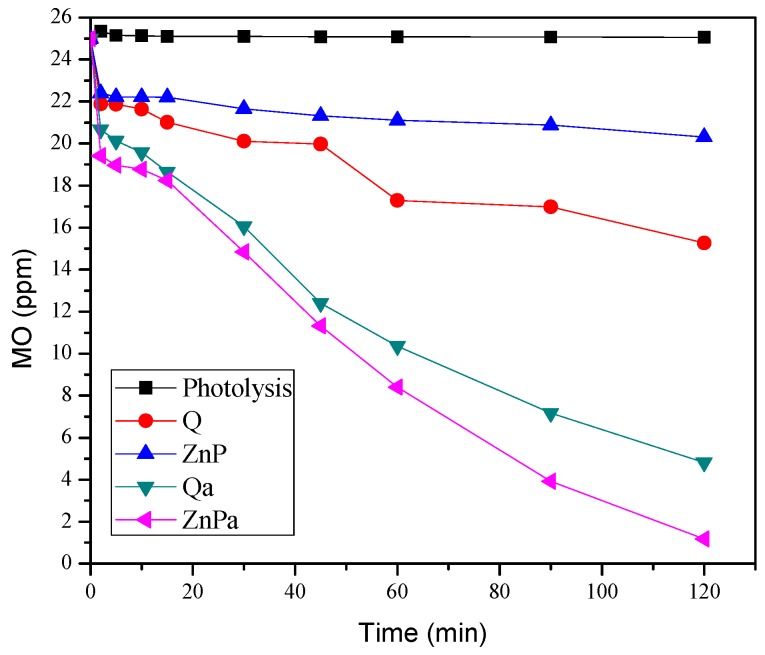
Evolution of the methyl orange concentration during its photodegradation over each one of the photocatalysts evaluated at different reaction time.

Figure 8, shows the MO concentration evolution with the photoreaction time, and as it can be observed, the highest degradation of the dye was achieved over the ZnPa catalyst.

As it was indicated in the experimental section, selected photocatalysts were also tested in the phenol and MO degradation reactions under UV-Visible and visible radiations, and the results are presented in Table 2. It can be observed that the degradation rate for MO over all the analyzed catalysts is higher than the observed in the case of phenol. This behavior can be due to a double sensitization phenomenon induced by the simultaneous presence of the sensitizing agent in the catalyst and the dye used as a substrate in the photoreaction system.

In the case of the photoreactions conducted under visible light, it is observed that all the evaluated catalysts are active under this radiation, thus, demonstrating the effectiveness of the dye sensitization treatment. It is also important to remark that, as expected, under visible light, the MO and phenol degradation rate decreases.

## 4. Conclusions

The dye sensitization is an effective method to obtain the lab prepared TiO_2_ catalysts active and effective in the treatment of water pollutants such as phenol and methyl orange by photocatalysis.

The effectiveness of the sensitization depends on the correct selection of the sensitizer and it is also influenced by the substrate to be degraded.

Thus, for one hand, Quinizarin is a good dye to prepare TiO_2_ sensitized materials effective for the degradation of methyl orange. However, on the other hand, the sensitization has a detrimental effect on the phenol degradation rate.

Finally, the photocatalysts based on TiO_2_-nanotubes sensitized with the dye Zinc protoporfirin are the most effective materials for the MO photodegradation.

## Figures and Tables

**Table 1:** Bandgap values and *S*_BET_ of the analyzed photocatalysts.

Sample	S_BET_ (m^2^/g)	Band gap, eV
LT	153	3.4
Q	213.6	3.2
ZnP	172.6	3.2
TNT	73	3.5
Qa	50.3	3.3
ZnPa	80.6	3.3

**Table 2:** Phenol and methyl orange (MO) degradation rate under different light radiation.

Photocatalysts	Irradiation	Degradation rate (mg L^−1^·s^−1^)	
		MO	Phenol
Q	UV-Vis	1.33 × 10^−3^	1.09 × 10^−4^
Q	Vis	1.10 × 10^−3^	1.80 × 10^−4^
ZnP	UV-Vis	4.31 × 10^−4^	1.05 × 10^−4^
ZnP	Vis	4.14 × 10^−4^	—
Qa	Vis	1.0 × 10^−3^	—
